# Computational modeling of apoptotic signaling pathways induced by cisplatin

**DOI:** 10.1186/1752-0509-6-122

**Published:** 2012-09-11

**Authors:** Ji-Young Hong, Geun-Hong Kim, Jun-Woo Kim, Soon-Sung Kwon, Eisuke F Sato, Kwang-Hyun Cho, Eun Bo Shim

**Affiliations:** 1 Department of Mechanical and Biomedical Engineering, Kangwon National University, 192-1, Hyoja 2-dong, Chuncheon, Gangwon-do, 200-701, Republic of Korea; 2 Department of Medical Biochemistry, Suzuka University of Medical Science, 3500-3 Minamitamagaki, Suzuka, mie 513-8670, Japan; 3 Department of Bio and Brain Engineering, Korea Advanced Institute of Science and Technology (KAIST), 291 Daehak-ro, Yuseong-gu, Daejeon, 305-701, Republic of Korea

**Keywords:** Apoptotic pathways, Cisplatin, Mathematical model

## Abstract

**Background:**

Apoptosis is an essential property of all higher organisms that involves extremely complex signaling pathways. Mathematical modeling provides a rigorous integrative approach for analyzing and understanding such intricate biological systems.

**Results:**

Here, we constructed a large-scale, literature-based model of apoptosis pathways responding to an external stimulus, cisplatin. Our model includes the key elements of three apoptotic pathways induced by cisplatin: death receptor-mediated, mitochondrial, and endoplasmic reticulum-stress pathways. We showed that cisplatin-induced apoptosis had dose- and time-dependent characteristics, and the level of apoptosis was saturated at higher concentrations of cisplatin. Simulated results demonstrated that the effect of the mitochondrial pathway on apoptosis was the strongest of the three pathways. The cross-talk effect among pathways accounted for approximately 25% of the total apoptosis level.

**Conclusions:**

Using this model, we revealed a novel mechanism by which cisplatin induces dose-dependent cell death. Our finding that the level of apoptosis was affected by not only cisplatin concentration, but also by cross talk among pathways provides *in silico* evidence for a functional impact of system-level characteristics of signaling pathways on apoptosis.

## Background

Cisplatin is an effective chemotherapeutic agent widely used in the treatment of cancer [1,2], but it has several side effects, including dose-dependent renal cell death and nephrotoxicity [3-6]. Uptake of cisplatin occurs mainly through the organic transporter pathway, and the kidney accumulates cisplatin to a greater degree than other organs. These events cause tubular damage and tubular dysfunction [5]. High concentrations of cisplatin lead to necrosis in proximal tubule cells, whereas lower concentrations induce apoptosis [7-10].

**Figure 1:**
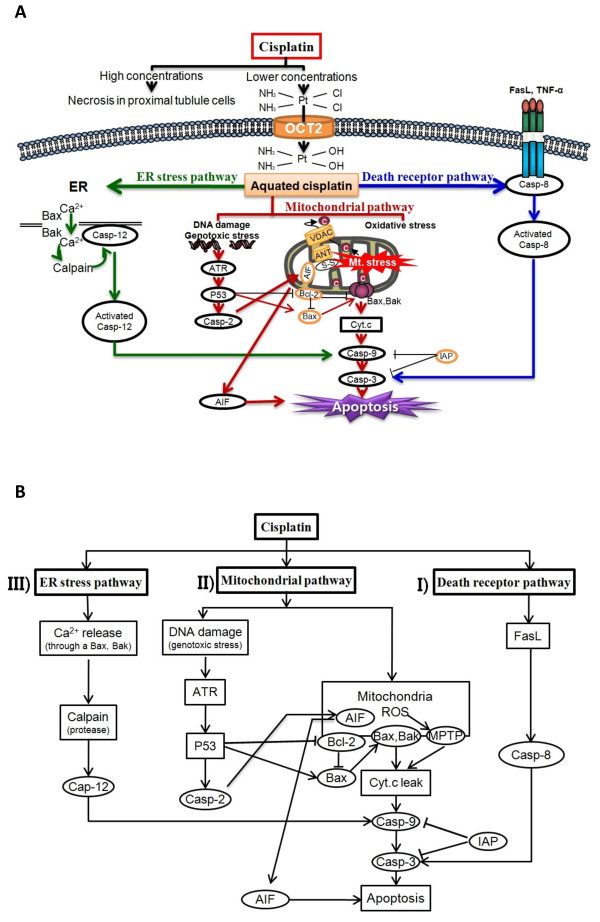
**Mathematical model of the apoptotic pathways induced by cisplatin.** (**A**) Schematic description of the apoptotic pathways induced by cisplatin. (**B**) Simplified model of apoptotic pathways. Cisplatin activates the three major pathways of apoptosis: i) the extrinsic pathway mediated by death receptors; ii) the intrinsic pathway centered on mitochondria; iii) the endoplasmic reticulum (ER)-stress pathway. Solid arrows denote chemical reactions or upregulation; those terminated by a bar denote inhibition or downregulation.

Recent studies have indicated that extranuclear events involving mitochondria [11], endoplasmic reticulum (ER) [12], and lysosomes [13] may be important for the induction of cellular apoptosis by cisplatin [12]. Apoptosis can also be triggered by extracellular death signals, deprivation of survival signals, and genetic damage [14]. Experimental observations [15-22] have shown that apoptosis occurs along three major pathways: i) an extrinsic pathway mediated by death receptors, ii) an intrinsic pathway centered on mitochondria, and iii) an ER-stress pathway (Figure 1A). 

Despite numerous experimental studies, the biological mechanism underlying the apoptotic effect of cisplatin is not yet completely understood. In particular, because cellular apoptosis is related to complicated and interactive signaling pathways and also depends on the cisplatin concentration [11], an experimental approach alone may not be cost effective for delineating the complex mechanism of cisplatin-induced apoptosis. Thus, a theoretical approach based on mathematical formulations can provide an alternative to complement experimental methods.

A variety of theoretical studies have investigated the effects of specific pathways or variables related to apoptosis. For example, Fussenegger et al. [23] presented a mechanistic mathematical model describing key elements of receptor-mediated and stress-induced caspase activation. Choi et al. [24] focused on the “slow induction plus fast switching” mechanism of caspase-3 using a simplified model. Apoptosis can also occur in response to activation of the mitochondrial apoptotic pathway [25]. Legewie et al. [26] proposed a mathematical model for the mitochondrial pathway of caspase activation, which is essential for induction of apoptosis by various stimuli, including cytotoxic stress. However, most previous models have focused on partial signaling pathways or proteins related to apoptosis, and no theoretical studies have examined an integrative model that includes all three major pathways of apoptotic signaling induced by cisplatin.

In this study, we constructed a large-scale, literature-based mathematical model to gain a systematic understanding of the biological mechanisms underlying cisplatin-induced apoptosis. Using the model, sequential signaling events, from the uptake of cisplatin to cellular apoptosis, were simulated. We then analyzed the characteristics of dose-dependent cellular apoptosis and cross-talk effects among the three apoptosis pathways through extensive simulations.

## Results and discussions

### Model structure

A schematic representation of the apoptotic signaling network described by our computational model is shown in Figure 1B. The model starts with the uptake of cisplatin into a cell by organic cation transporter 2 (OCT2). After entering the cell, cisplatin is aquated into a highly reactive form that can bind to and induce modification of various molecules [27,28]. Cisplatin activates the three major pathways of apoptosis: i) the extrinsic pathway mediated by death receptors, ii) the intrinsic pathway centered on mitochondria, and iii) the ER stress pathway [3,4,6,12].

First, in the extrinsic pathway, binding of the death receptors by ligands at the plasma membrane leads to the recruitment and activation of caspase-8, which further activates downstream caspases to induce apoptosis, because the expression levels of FasL, Fas, and tumor necrosis factor-alpha (TNF-α) increase in response to cisplatin [29-31]. Second, the mitochondrial pathway is the major apoptotic pathway involved in the nephrotoxicity of cisplatin. Cisplatin induces DNA damage, which activates ataxia telangiectasia and Rad-3-related (ATR), resulting in the phosphorylation and activation of p53. Then, p53 activates caspase-2 to elicit apoptosis-inducing factor (AIF) release from mitochondria and subsequent caspase-independent apoptosis. The capacity of p53 to directly activate Bax to permeabilize mitochondria permits an uninterrupted pathway leading, e.g., from DNA damage to the mitochondrial release of cytochrome *c*, caspase activation, and apoptosis [3,32-34]. In addition, cisplatin induces mitochondrial dysfunction and increases reactive oxygen species (ROS) production via the disrupted respiratory chain [35]. Oxidative injury of mitochondrial function and mtDNA (mitochondrial DNA) in the kidney are early events elicited by cisplatin [36]. Uncontrollable production of ROS triggers opening of mitochondrial permeability transition pores (MPTP) and induces apoptosis through the release of cytochrome *c* from mitochondria into the cytosol [37-40]. Third, in the ER stress pathway, increased cytosolic calcium and calpain activation are early events in cisplatin-induced apoptosis [41]. Calpain is a protease responsible for activation of caspase-12 [42], which is localized at the cytosolic face of the ER [43]. Cisplatin induces apoptosis in the absence of DNA damage, and the ER is likely its nonnuclear target [12].

### Baseline results of cisplatin-induced apoptosis

**Figure 2:**
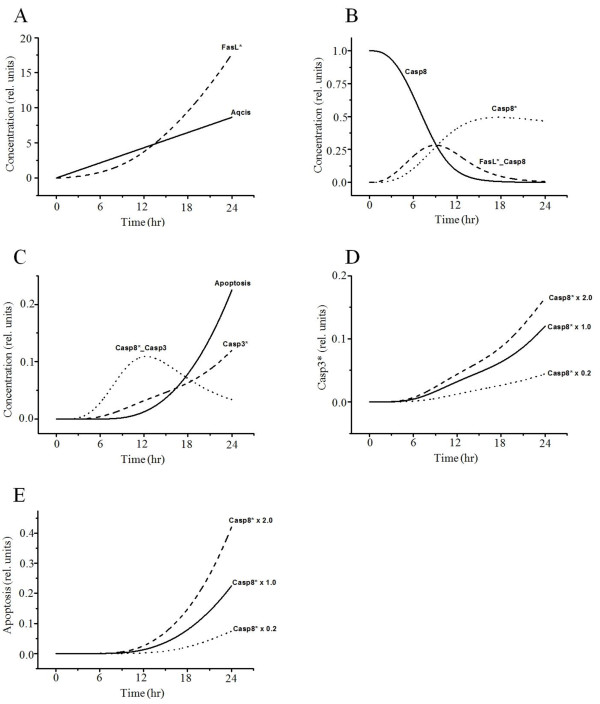
**Simulated results of the variables related to the death receptor pathway.** (**A**) Cisplatin becomes aquated and toxic after its entry into cells, thus activating FasL. Aqcis, aquated cisplatin; FasL*, activated FasL. (**B**) Activated FasL binds caspase-8 and activates the initiator caspase-8. FasL*_Casp8, binding of FasL* and Casp-8; Casp8*, activated caspase-8; Casp8, procaspase-8. (**C**) The activated caspase-8 leads directly to the activation of downstream caspase-3 and causes apoptosis. Casp8*_casp3, binding of Casp8* and casp3; Casp3*, activated caspase-3. Interaction between activated caspase-8 and caspase-3 activation (**D**) or apoptosis (**E**). Casp8*  × 1.0, activated caspase-8 at baseline value; Casp8*  × 2.0, activated caspase-8 increase by twofold; Casp8*  ×  0.2, activated caspase-8 decrease by fivefold.

Figure 2 presents time series of the equation variables related to the death receptor pathway. The proteins of the TNF-α receptor family, including Fas ligand (FasL) and TNF receptor 1 (TNFR 1), play important roles in apoptotic cell death. FasL activates the initiator caspase-8 (Figure 2A, B), and then activated caspase-8 leads directly to the activation of downstream caspase-3, causing apoptosis (Figure 2C) [29]. Similar to previous experiments of cisplatin-induced cell death [44,45]; all variable curves were plotted until 24 h after uptake of cisplatin. To evaluate the interaction between activated caspase-8 and caspase-3 activation or apoptosis, we tested our mathematical model for different levels of activated caspase-8 expression. Less-activated caspase-8 implies reduced caspase-3 activation and apoptosis (Figure 2D, E). Activated caspase-8 initiates a caspase cascade by processing the effectors caspase-3, -6, and −7, which in turn cleave many protein substrates. These results showed similar patterns to those reported in the literature [29], indicating that caspase-8 is a major initiator caspase in death receptor signaling. 

**Figure 3:**
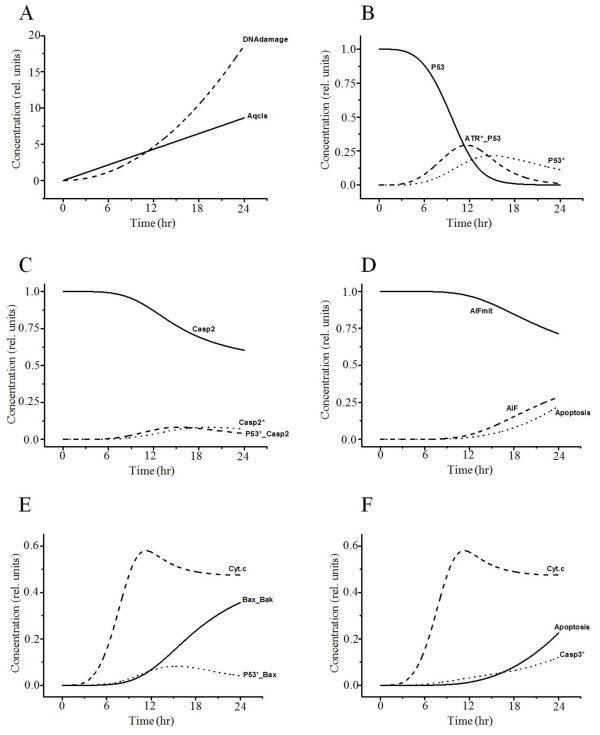
**Simulated results of the variables related to the mitochondrial pathway by DNA damage.** (**A**) Cisplatin induces DNA damage. (**B**) DNA damage activates ATR and p53. ATR*_p53, binding of ATR* and p53. (**C**) P53 then induces activation of caspase-2. p53*_Casp2, binding of p53* and casp2, (**D**) leading to AIF release from mitochondria and subsequent caspase-independent apoptosis. AIFmit, AIF in mitochondria; AIF, leaked AIF in the cytosol. (**E**, **F**) On the other hand, apoptosis induced by p53 binding with Bax is dependent on Bax/Bak, which induces cytochrome *c* release. Cyt.*c*, leaked cytochrome *c* in the cytosol; p53*_Bax, binding of p53* and Bax; Bax_Bak, binding of Bax and Bak.

**Figure 4:**
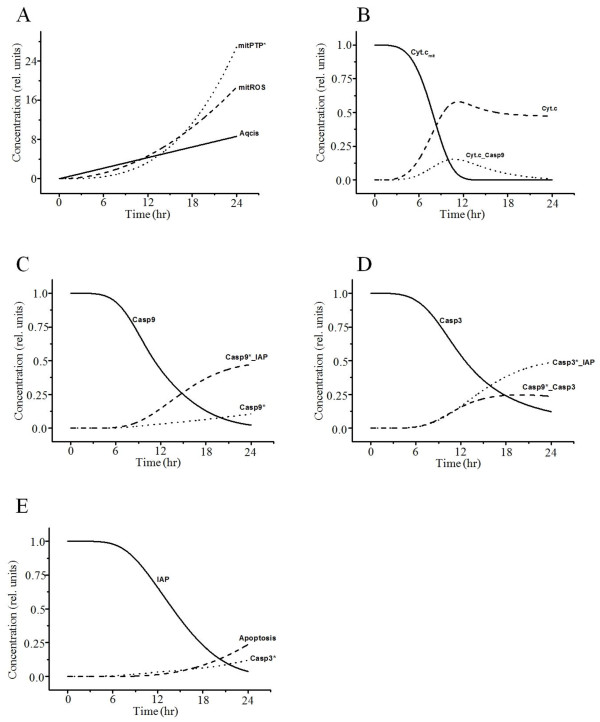
**Simulated results of the variables related to the mitochondrial pathway by oxidative stress.** (**A**) Increased ROS in mitochondria by cisplatin induces MPTP. Aqcis, aquated cisplatin; mitROS, increased ROS in mitochondria; mitPTP*, opened mitochondrial permeability transition pore. (**B**) MPTP induces cytochrome *c* release, which activates caspase-9. Cyt.*c*, cytochrome *c* in cytosol; Cyt.*c*_mit_, cytochrome *c* in mitochondria; Cyt.*c*_Casp9, binding of Cyt.*c* and Casp9. (**C**, **D**) IAP inhibits activated caspase-9 and −3, which induce apoptosis. Casp9*_IAP, binding of Casp9* and IAP; Casp3*_IAP, binding of Casp3* and IAP; Casp9*_Casp3, binding of Casp9* and Casp3. Activated caspase-9 subsequently initiates caspase-3 activation (**D**) and induces apoptosis (**E**).

Time-dependent curves of the variables related to the mitochondrial pathway of apoptosis due to mtDNA damage and oxidative stress are plotted in Figures 3 and 4, respectively. The p53 activated by DNA damage activates caspase-2 to induce AIF (Figure 3A–D) and the Bax-mediated mitochondrial pathway (Figure 3E, F) for apoptosis [46,47]. Cytochrome *c* is released from mitochondria into the cytosol after activation of Bax insertion into mitochondrial membranes and cytochrome *c* additionally activates caspase-3 (Figure 3E, F) [48,49]. AIF is another protein released from mitochondria into the cytosol that causes apoptosis in a caspase-independent manner by inducing DNA damage in the nucleus (Figure 3D) [50,51]. Cisplatin also induces ROS production via the disrupted respiratory chain [35]. Overproduction of ROS in mitochondria induces MPTP opening, and cytochrome *c* is released from mitochondria through MPTP in the early stages of apoptosis (Figure 4A, B) [37]. Cytosolic cytochrome *c* (Figure 4B) activates caspase-9 (Figure 4C), which triggers caspase-3 (Figure 4D) and causes consequent apoptosis (Figure 4E). 

**Figure 5:**
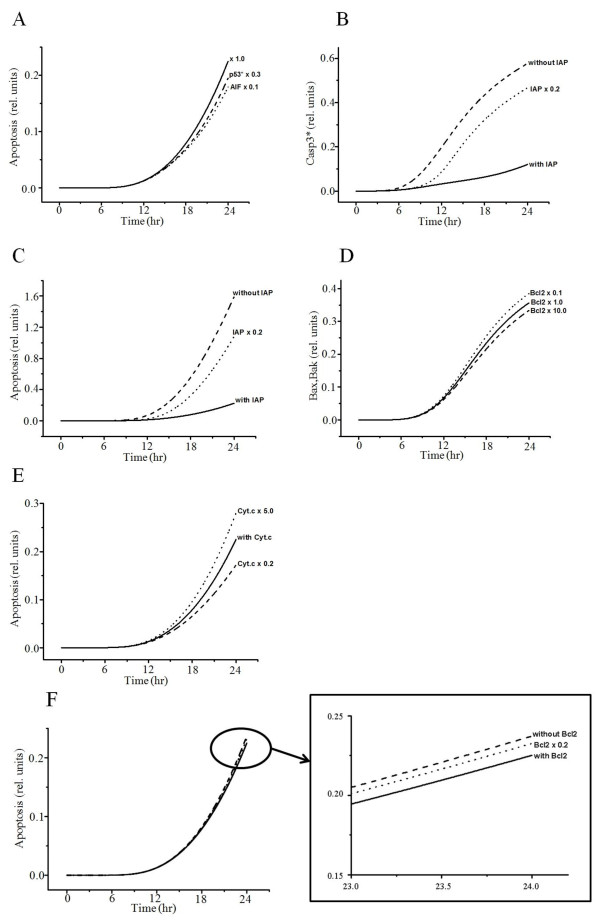
**Interaction between apoptosis and AIF, p53 activation, IAP, Bcl-2, or Cyt.*c*.** (**A**) Interaction between apoptosis and p53* or IAP. Inhibition of p53 and AIF shows a decrease in apoptosis.  × 1.0, baseline value; p53*  × 0.3, activated p53 decrease by thirtyfold; AIF  ×  0.1, AIF decrease by tenfold. (**B**) Interaction between Casp3* and IAP without IAP, IAP  = 0; with IAP, IAP at baseline value; IAP  × 0.2, IAP decrease by twentyfold. (**C**) Interaction between apoptosis and IAP. Bcl-2 impairs activation of Bax/Bak. (**D**) Interaction between Bax_Bak and Bcl-2. Bcl-2  × 1.0, Bcl-2 at baseline value; Bcl-2  × 0.1, Bcl-2 decrease by tenfold; Bcl-2  × 10.0, Bcl-2 increase by tenfold. (**E**) Interaction between apoptosis and Cyt.*c*. with Cyt.*c*, Cyt.*c* at baseline value; Cyt.*c*  ×  5.0, Cyt.*c* increase by fivefold; Cyt.*c*  ×  0.2, Cyt.*c* decrease by fivefold. (**F**) Interaction between apoptosis and Bcl-2 without Bcl-2, Bcl-2  = 0; with Bcl-2, Bcl-2 at baseline value; Bcl-2  × 0.2, IAP decrease by twentyfold.

When we inhibited p53 or AIF, the simulated graphs showed similar patterns to previously reported experimental results [32]: inhibition of p53 or AIF provides protection against cisplatin-induced apoptosis (Figure 5A). Reduced inhibitor of apoptosis protein (IAP) increases the concentration of activated caspase-3, increasing the probability of apoptosis (Figure 5B, C). The anti-apoptotic protein Bcl-2 impairs the activation of Bax/Bak (Figure 5D), thereby maintaining mitochondrial membrane integrity and reducing cytochrome *c* leakage, which results in a decrease in the probability of apoptosis (Figure 5E). However, a reduction in Bcl-2 expression slightly increases the probability of apoptosis (Figure 5F). 

**Figure 6:**
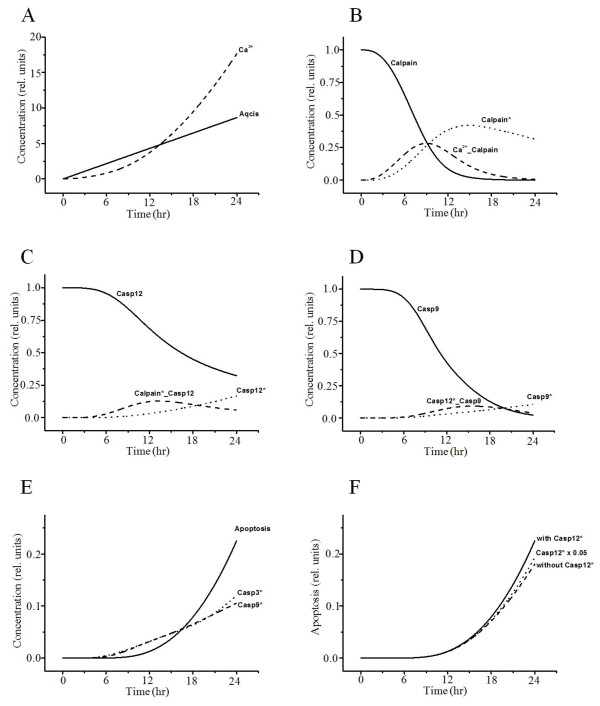
**Simulated results of the variables related to the ER-stress pathway.** (**A**) Cisplatin induces ER stress and leads to release of Ca^2+^ from the ER. Ca^2+^ was leaked from the ER. Ca^2+^ activates calpain (**B**), which cleaves procaspase-12 to mature caspase-12 (**C**). Ca^2+^_Calpain, binding of Ca^2+^ and Calpain; Calpain*_Casp12, binding of Calpain* and Casp12. (**D**) Activated caspase-12 initiates caspase-9. Casp12*_Casp9, binding of Casp12* and Casp9. (**E**) Activated caspase-9 and −3 cause apoptosis. (**F**) Interaction between apoptosis and activated caspase-12. The simulation is based on a model with (× 1.0) or without activated caspase-12. Casp12*  × 0.05, Casp12* decrease by twofold.

Accumulation of excessive proteins or disruption of calcium homeostasis in the ER can cause apoptosis due to ER stress [42,52]. Transient curves of the variables related to the ER-stress pathway are presented in Figure 6. ER stress causes conformational changes and/or oligomerization of pro-apoptotic Bak and Bax at the ER membrane [53], leading to release of Ca^2+^ from the ER (Figure 6A). Ca^2+^ then activates calpain in the cytosol, which cleaves procaspase-12 to mature caspase-12 in the ER (Figure 6B, C) [54]. Activated caspase-12 then initiates a caspase cascade through cleavage of procaspase-9 and thus causes apoptosis (Figure 6D, E) [55]. Inhibition of caspase-12 activated by cisplatin induces a slight decrease in the probability of apoptosis (Figure 6F). These results suggest that ER stress and consequent activation of caspase-12 play a role in cisplatin-induced nephrotoxicity, but their effect on apoptosis was not remarkable. 

### Simulation of dose-dependent cell death caused by cisplatin

**Figure 7:**
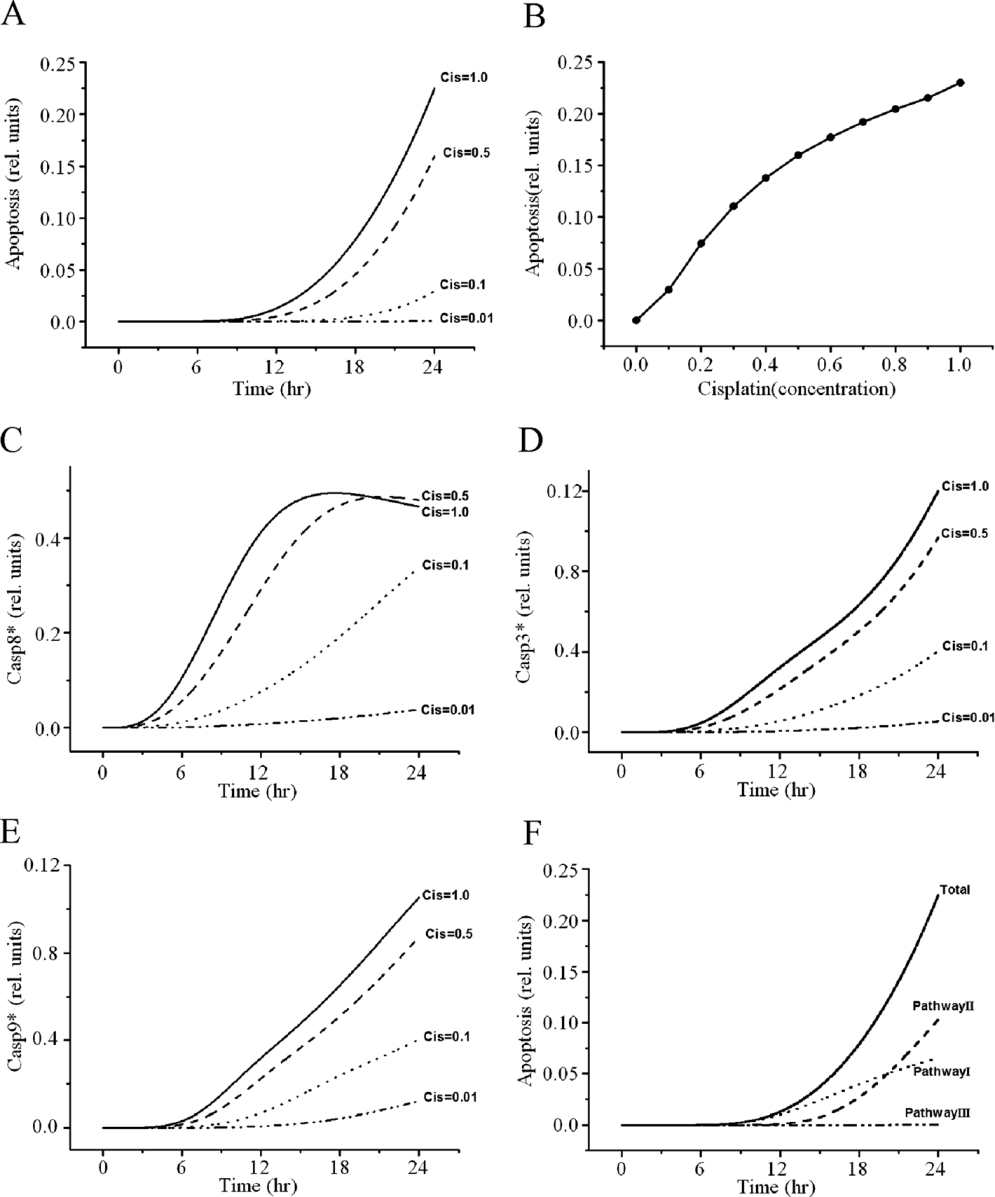
**Effect of cisplatin concentration on caspase activity and apoptosis.** (**A**, **B**) Relationship between apoptosis and various concentrations of cisplatin. Cisplatin induces dose-dependent apoptosis. **C**, **D**, and **E** show the activation of caspase-8, -3, and −9, respectively, with increasing doses of cisplatin for 24 h. (**F**) Effect of apoptosis by each pathway.

Cisplatin has been shown to induce time- and dose-dependent cell death [56,57]. Although the maximum concentration of cisplatin was set to 1 in our model, we simulated the apoptosis level in response to various concentrations of cisplatin on a timescale. The increase in the level of apoptosis was dependent on the concentration of cisplatin (Figure 7A, B, Table 1). Cisplatin-induced apoptosis was further confirmed by the activities of caspase-9, -8, and −3 (Figure 2C, E and Figure 6E). Figure 7C–E indicates that increasing doses of cisplatin can cause significant activation of caspase-8, -3, and −9. Although the level of apoptosis increased linearly at lower concentrations of cisplatin, it exhibited a nearly saturated curve at higher concentrations (Figure 7B). A previous experimental study [55] also observed a saturated probability of apoptosis beyond a threshold concentration of cisplatin. 

**Table 1:** Apoptotic variation in response to cisplatin concentration and pathway

**Cisplatin concentration**	**Apoptosis level**
0.0	0.0
0.0001	0.0
0.001	5E-6
0.01	4.67E-4
0.1	2.96E-2
0.5	0.16
1.0	0.23

(A) Apoptosis level in various concentrations of cisplatin. (B) Apoptosis level in each pathway.

### Analysis of cross-talk effects of apoptotic signaling pathways

To evaluate the relative contribution of each signaling pathway to apoptosis, we first simulated three test cases in which only one pathway among the three was activated, with the other two pathways fully inactivated (Figure 7F, Table 2). According to the results, the mitochondrial pathway made the greatest contribution to the level of apoptosis (46%), whereas the contribution of the ER stress pathway (0.08%) was negligible. The contribution of the death-receptor pathway was about 29%. The sum of the contributions of the three pathways was about 75% and lower than that of the whole model including all three pathways (Table 2). Thus, we hypothesize that cross talk among pathways accounts for 25% of the contribution to the level of apoptosis. This cross-talk portion includes the effects of three possible interactions: i) between death receptor and ER-stress pathways, ii) between death receptor and mitochondrial pathways, and iii) between ER-stress and mitochondrial pathways.

**Table 2:** Apoptosis level in each pathway

**Pathway**	**Apoptosis level**
Total	0.22512 (100%)
Pathway I	0.065424 (29.1%)
Pathway II	0.103267 (45.9%)
Pathway III	1.83E-4 (0.08%)
Cross talk effect	0.0561 (24.9%)

Despite substantial progress in understanding the biological mechanisms of cisplatin-induced apoptosis, several questions remain. In particular, complicated molecular interactions, such as cross talk among apoptotic pathways, are not fully understood. In this study, we provided a mechanism-based mathematical model to gain a comprehensive system-level understanding of apoptosis induced by cisplatin. Because our model simulation can be cost-effectively repeated for many different sets of conditions, it provides a useful method for examining complex system behavior and for guiding experimental design.

Although many mathematical models have analyzed apoptosis, they have been limited to partial signaling pathways or to proteins related to apoptosis. No theoretical studies have proposed an integrative model that includes all three major pathways of apoptotic signaling induced by cisplatin. Therefore, we aimed to systematically analyze sequential molecular events of the apoptotic signaling process, from the uptake of cisplatin to cross-talk effects among pathways and ultimately to apoptosis.

We first conducted baseline simulations of cisplatin-induced apoptosis and compared the results to previous studies. Our simulations reproduced similar patterns to experimental data and available information from the literature (Figure 2A–C, Figure 3, Figure 4, and Figure 6A–E). We then conducted parametric studies to delineate the effects of critical signaling molecules on apoptosis. To evaluate the role of caspase-8 as a major initiator of death receptor signaling, we simulated variation in activated caspase-3 and the level of apoptosis based on the level of activated capase-8 (Figure 2D, E), demonstrating a remarkable increase in the level of apoptosis at higher concentrations of activated capase-8. In the mitochondrial pathway of apoptosis, inhibition of p53 or the level of AIF decreased apoptosis (Figure 5A).

We also showed that cisplatin induces dose-dependent characteristics of apoptosis (Figure 7A, B, Table 1), but the apoptosis level was saturated at higher concentrations of cisplatin, similar to a previous experiment [57]. Apoptosis with respect to cisplatin concentration was also affected by cross talk among pathways (Figure 5 and Figure 7). To evaluate the contribution of each pathway to apoptosis, we used a simulation protocol in which only one pathway was activated while the other two pathways were inactivated. According to the results, the effect of the mitochondrial signaling pathway on apoptosis was largest (46%), whereas the effect of the ER-stress pathway was negligible (0.08%). The contribution of the death-receptor pathway was about 29%. Using mitochondrial DNA-depleted p^0^ cells, Hara et al. [58] demonstrated that mitochondria play a critical role in apoptosis induced by cisplatin and that mitochondrial DNA is a potential target for cisplatin. Also, mitochondria-enriched renal cells in the proximal tubule are critical sites for the occurrence of side effects of cisplatin [59,60]. These experimental results suggest that the mitochondrial pathway is the major apoptotic pathway involved in the nephrotoxicity of cisplatin.

The sum of the contributions of all three pathways to the total apoptosis level was about 75%, whereas 25% of the total apoptosis level was attributable to cross talk among pathways. These results indicated that the level of apoptosis was affected not only by cisplatin concentration, but also by cross talk, i.e., complex interactions of molecular components among pathways.

## Conclusions

Although we applied a systematic analysis of the apoptotic mechanisms induced by cisplatin using a comprehensive model, the approach had several limitations. First, parameter values in the model were based on values obtained from the literature or adjusted manually when sufficient information could not be obtained from the literature or experimental studies. Second, as in previous studies [61,62], we simply set the initial conditions as 0 or 1. However, as was demonstrated by the sensitivity analyses and perturbation test of the parameters and initial conditions (described in the “Methods” section), these limitations were not expected to greatly alter the main findings of this study. In a future study, we will design a smaller model that focuses on the main apoptotic pathway by cisplatin and will obtain parameters by fitting the model to experimental data.

## Methods

### Mathematical model

Mathematical descriptions of interactions of the mechanistic parts of the pathways were modeled based on biochemical reaction equations. The signal transduction network of apoptotic reactions is represented by ordinary differential equations (ODEs). This network leads to a set of 44 ODEs with 24 reaction partners (Table 3 and Table 4). In the reactions, the effects of synthesis and degradation are not taken into account. The differential equations in the modeling of the signaling pathways were solved using MATLAB library functions. 

**Table 3:** Descriptions of reactions

**Descriptions of reactions**	
I.	II-2.
$\begin{array}{c} Cisplatin+OCT2\;\overset{k_{1}^{+}}{\to }\;Aquated\;Cis\\ Aquated\;Cis+FasL\;\overset{k_{32}^{+}}{\to }FasL^{*}\\ FasL^{*}+\;Casp8\overset{k_{33}^{+}}{\underset{k_{33}^{-}}{\;⇄}}\;FasL^{*}\cdot Casp8\overset{k_{34}^{+}}{\to }Casp8^{*}\\ Casp8^{*}+Casp3\;\overset{k_{35}^{+}}{\underset{k_{35}^{-}}{⇄}}Casp8^{*}\cdot Casp3\overset{k_{36}^{+}}{\to }\\ Casp8^{*}+Casp3^{*}\overset{k_{37}^{+}}{\to }Apop\\ Casp3^{*}+IAP\overset{k_{13}^{+}}{\underset{k_{13}^{-}}{⇄}}\;Casp3^{*}\cdot IAP \end{array}$	$\begin{array}{c} Cisplatin\;+\;OCT2\;\overset{k_{1}^{+}}{\to }\;Aquated\;Cis\\ Aquated\;Cis+Mit\overset{k_{29}^{+}}{\to }{}_{\mathrm{mit}}Ros\\ {}_{\mathrm{mit}}Ros\;+\;{}_{\mathrm{mit}}PTP\overset{k_{30}^{+}}{\to }{}_{\mathrm{mit}}PTP^{*}\\ \;{}_{\mathrm{mit}}PTP^{*}+Cytc{}_{\mathrm{mit}}\overset{k_{31}^{+}}{\to }{}_{\mathrm{mit}}PTP^{*}+Cytc\\ Cytc+Casp9\;\overset{k_{27}^{+}}{\underset{k_{27}^{-}}{⇄}}\;Cytc\cdot Casp9\overset{k_{28}^{+}}{\to }Casp9^{*}\\ Casp9^{*}+Casp3\;\overset{k_{9}^{+}}{\underset{k_{9}^{-}}{⇄}}\;Casp9^{*}\cdot Casp3\overset{k_{10}^{+}}{\to }\\ \;Casp9^{*}+Casp3^{*}\overset{k_{11}^{+}}{\to }Apop\\ Casp9^{*}+IAP\overset{k_{12}^{+}}{\underset{k_{12}^{-}}{⇄}}Casp9^{*}\cdot IAP\\ \;Casp3^{*}+IAP\overset{k_{13}^{+}}{\underset{k_{13}^{-}}{⇄}}Casp3^{*}\cdot \;IAP \end{array}$
II-1.	III.
$\begin{array}{c} Cisplatin+OCT2\overset{k_{1}^{+}}{\to }Aquated\;Cis\\ Aquated\;Cis+DNA\overset{k_{14}^{+}}{\to }DNA_{\mathrm{damage}}\\ DNA_{\mathrm{damage}}+ATR\overset{k_{15}^{+}}{\to }ATR^{*}\\ ATR^{*}+P53\overset{k_{16}^{+}}{\underset{k_{16}^{-}}{⇄}}ATR^{*}\cdot P53\overset{k_{17}^{+}}{\to }P53^{*}\\ P53^{*}+Casp2\overset{k_{18}^{+}}{\underset{k_{18}^{-}}{⇄}}P53^{*}\cdot Casp2\overset{k_{19}^{+}}{\to }P53^{*}+Casp2^{*}\\ Casp2^{*}+AIF_{\mathrm{mit}}\overset{k_{20}^{+}}{\to }Casp2^{*}+AIF\overset{k_{21}^{+}}{\to }Apop\\ P53^{*}+Bax\overset{k_{22}^{+}}{\underset{k_{22}^{-}}{⇄}}P53^{*}\cdot Bax\overset{k_{23}^{+}}{\to }Bax\cdot Bak\\ Bcl2+Bax\overset{k_{24}^{+}}{\underset{k_{24}^{-}}{⇄}}Bcl2\cdot Bax\\ P53^{*}+Bcl2\overset{k_{25}^{+}}{\underset{k_{25}^{-}}{⇄}}P53^{*}\cdot Bcl2\\ Bax\cdot Bak+Cytc_{\mathrm{mit}}\overset{k_{26}^{+}}{\to }Bax\cdot Bak+Cytc\\ Cytc+Casp9\overset{k_{27}^{+}}{\underset{k_{27}^{-}}{⇄}}Cytc\cdot Casp9\overset{k_{28}^{+}}{\to }Casp9^{*}\\ Casp9^{*}+Casp3\overset{k_{9}^{+}}{\underset{k_{9}^{-}}{⇄}}Casp9^{*}\cdot Casp3\overset{k_{10}^{+}}{\to }\\ Casp9^{*}+Casp3^{*}\overset{k_{11}^{+}}{\to }Apop\\ Casp9^{*}+IAP\overset{k_{12}^{+}}{\underset{k_{12}^{-}}{⇄}}Casp9^{*}\cdot IAP\\ Casp3^{*}+IAP\overset{k_{13}^{+}}{\underset{k_{13}^{-}}{⇄}}Casp3^{*}\cdot IAP \end{array}$	$\begin{array}{c} Cisplatin+OCT2\overset{k_{1}^{+}}{\to }Aquated\;Cis\\ Aquated\;Cis+ER\overset{k_{2}^{+}}{\to }Ca^{2+}\\ Ca^{2+}+Calpain\overset{k_{3}^{+}}{\underset{k_{3}^{-}}{⇄}}Ca^{2+}\cdot Calpain\overset{k_{4}^{+}}{\to }Calpain^{*}\\ Calpain^{*}+Casp12\overset{k_{5}^{+}}{\underset{k_{5}^{-}}{⇄}}Calpain^{*}\cdot Casp12\overset{k_{6}^{+}}{\to }Casp12^{*}\\ Casp12^{*}+Casp9\overset{k_{7}^{+}}{\underset{k_{7}^{-}}{⇄}}Casp12^{*}\cdot Casp9\overset{k_{8}^{+}}{\to }Casp12^{*}+Casp9^{*}\\ Casp9^{*}+Casp3\overset{k_{9}^{+}}{\underset{k_{9}^{-}}{⇄}}Casp9^{*}\cdot Casp3\overset{k_{10}^{+}}{\to }Casp9^{*}+Casp3^{*}\overset{k_{11}^{+}}{\to }Apop\\ Casp9^{*}+IAP\overset{k_{12}^{+}}{\underset{k_{12}^{-}}{⇄}}Casp9^{*}\cdot IAP\\ Casp3^{*}+IAP\overset{k_{13}^{+}}{\underset{k_{13}^{-}}{⇄}}Casp3^{*}\cdot IAP \end{array}$

k^+^, forward rate constant of reaction; k^–^, reverse rate constant of reaction; Casp12*, activated caspase-12.

**Table 4:** Ordinary differential equation

**Ordinary differential equations**	
$\begin{array}{c} d\left[Aqcis\right]/dt=k_{1}^{+}\left[Cis\right]\left[OCT2\right]\\ d\left[Ca^{2+}\right]/dt=k_{2}^{+}\left[Aqcis\right]\left[ER\right]-k_{3}^{+}\left[Ca^{2+}\right]\left[Calpain\right]+k_{3}^{-}\left[Ca^{2+}\cdot Calpain\right]\\ d\left[Calpain\right]/dt=-k_{3}^{+}\left[Ca^{2+}\right]\left[Calpain\right]+k_{3}^{-}\left[Ca^{2+}\cdot Calpain\right]\\ d\left[Ca^{2+}\cdot Calpain\right]/d\text{t}=k_{3}^{+}\left[Ca^{2+}\right]\left[Calpain\right]+k_{3}^{-}\left[Ca^{2+}\cdot Calpain\right]\\ \;-k_{4}^{+}\left[Ca^{2+}\cdot Calpain\right]\\ d\left[Calpain^{*}\right]/dt=k_{4}^{+}\left[Ca^{2+}\cdot Calpain\right]-k_{5}^{+}\left[Calpain^{*}\right]\left[Casp12\right]\\ \;+k_{5}^{-}\left[Calpain^{*}\cdot Casp12\right]\\ d\left[Casp12\right]/dt=-k_{5}^{+}\left[Calpain^{*}\right]\left[Casp12\right]+k_{5}^{-}\left[Calpain^{*}\cdot Casp12\right]\\ d\left[Calpain^{*}\cdot Casp12\right]/dt=k_{5}^{+}\left[Calpain^{*}\right]\left[Casp12\right]\\ \;-k_{5}^{-}\left[Calpain^{*}\cdot Casp12\right]-k_{6}^{+}\left[Calpain^{*}\cdot Casp12\right]\\ d\left[Casp12^{*}\right]/dt=k_{6}^{+}\left[Calpain^{*}\cdot Casp12\right]-k_{7}^{+}\left[Casp12^{*}\right]\left[Casp9\right]\\ \;+k_{7}^{-}\left[Casp12^{*}\cdot Casp9\right]\\ d\left[Casp9\right]/dt=-k_{7}^{+}\left[Casp12^{*}\right]\left[Casp9\right]+k_{7}^{-}\left[Casp12^{*}\cdot Casp9\right]\\ \;-k_{27}^{+}\left[Cytc\right]\left[Casp9\right]+k_{27}^{-}\left[Cytc\cdot Casp9\right]\\ d\left[Casp12^{*}\cdot Casp9\right]/dt=k_{7}^{+}\left[Casp12^{*}\right]\left[Casp9\right]-k_{7}^{-}\left[Casp12^{*}\cdot Casp9\right]\\ \;-k_{8}^{+}\left[Casp12^{*}\cdot Casp9\right]\\ d\left[Casp9^{*}\right]/dt=k_{8}^{+}\left[Casp12^{*}\cdot Casp9\right]+k_{9}^{-}\left[Casp9^{*}\cdot Casp3\right]\\ \;-k_{9}^{+}\left[Casp9^{*}\right]\left[Casp3\right]-k_{12}^{+}\left[Casp9^{*}\right]\left[IAP\right]+k_{12}^{-}\left[Casp9^{*}\cdot IAP\right]\\ \;+k_{28}^{+}\left[Cytc\cdot Casp9\right] \end{array}$	$\begin{array}{c} d\left[ATR^{*}\right]/dt=k_{15}^{+}\left[DNA_{damage}\right]\left[ATR\right]-k_{16}^{+}\left[ATR^{*}\right]\left[P53\right]+k_{16}^{-}\left[ATR^{*}\cdot P53\right]\\ d\left[P53\right]/dt=k_{16}^{+}\left[ATR^{*}\right]\left[P53\right]+k_{16}^{-}\left[ATR^{*}\cdot P53\right]\\ d\left[ATR^{*}\cdot P53\right]/dt=k_{16}^{+}\left[ATR^{*}\right]\left[P53\right]-k_{16}^{-}\left[ATR^{*}\cdot P53\right]-k_{17}^{+}\left[ATR^{*}\cdot P53\right]\\ d\left[P53^{*}\right]/dt=k_{17}^{+}\left[ATR^{*}\cdot P53\right]+k_{18}^{-}\left[P53^{*}\cdot Casp2\right]-k_{18}^{+}\left[P53^{*}\right]\left[Casp2\right]\\ \;-k_{22}^{+}\left[P53^{*}\right]\left[Bax\right]+k_{22}^{-}\left[P53^{*}\cdot Bax\right]-k_{25}^{-}\left[P53^{*}\right]\left[Bcl2\right]+k_{25}^{-}\left[P53^{*}\cdot Bcl2\right]\\ d\left[Casp2\right]/dt=-k_{18}^{+}\left[P53\right]\left[Casp2\right]+k_{18}^{-}\left[P53^{*}\cdot Casp2\right]\\ d\left[P53^{*}\cdot Casp2\right]/dt=k_{18}^{+}\left[P53^{*}\right]\left[Casp2\right]-k_{18}^{-}\left[P53^{*}\cdot Casp2\right]-k_{18}^{-}\left[P53^{*}\cdot Casp2\right]\\ d\left[Casp2\right]/dt=k_{19}^{+}\left[P53\right]\left[Casp2\right]-k_{20}^{+}\left[Casp2^{*}\right]\left[AIF_{mit}\right]\\ d\left[AIF_{mit}\right]/dt=-k_{20}^{+}\left[Casp2^{*}\right]\left[AIF_{mit}\right]\\ d\left[AIF\right]/dt=k_{20}^{+}\left[Casp2^{*}\right]\left[AIF_{mit}\right]\\ d\left[Bax\right]/dt=-k_{22}^{+}\left[P53^{*}\right]\left[Bax\right]+k_{22}^{-}\left[P53^{*}\cdot Bax\right] \end{array}$
$\begin{array}{c} d\left[Casp3^{*}\right]/dt=k_{10}^{+}\left[Casp9^{*}\cdot Casp3\right]+k_{36}^{+}\left[Casp8^{*}\cdot Casp3\right]\\ \;-k_{13}^{+}\left[Casp3^{*}\right]\left[IAP\right]+k_{13}^{-}\left[Casp3^{*}\cdot IAP\right]\\ d\left[IAP\right]/dt=-k_{12}^{+}\left[Casp9^{*}\right]\left[IAP\right]+k_{12}^{-}\left[Casp9^{*}\cdot IAP\right]\\ \;-k_{13}^{+}\left[Casp3^{*}\right]\left[IAP\right]+k_{13}^{-}\left[Casp3^{*}\cdot IAP\right]\\ d\left[Casp9^{*}\cdot IAP\right]/dt=k_{12}^{+}\left[Casp9^{*}\right]\left[IAP\right]-k_{12}^{-}\left[Casp9^{*}\cdot IAP\right]\\ d\left[Casp3^{*}\cdot IAP\right]/dt=k_{13}^{+}\left[Casp3^{*}\right]\left[IAP\right]-k_{13}^{-}\left[Casp3^{*}\cdot IAP\right]\\ d\left[DNA_{damage}\right]/dt=k_{14}^{+}\left[Agcis\right]\left[DNA\right] \end{array}$	$\begin{array}{c} d\left[P53^{*}\cdot Bax\right]/dt=k_{22}^{+}\left[P53^{*}\right]\left[Bax\right]-k_{22}^{-}\left[P53^{*}\cdot Bax\right]-k_{23}^{-}\left[P53^{*}\cdot Bax\right]\\ d\left[Bax\cdot Bak\right]/dt=k_{23}^{+}\left[P53^{*}\cdot Bax\right]\\ d\left[Bcl2\right]/dt=-k_{25}^{+}\left[P53^{*}\right]\left[Bcl2\right]+k_{25}^{-}\left[P53^{*}\cdot Bcl2\right]\\ \;-k_{24}^{+}\left[Bcl2\right]\left[Bax\right]+k_{24}^{-}\left[Bcl2\cdot Bax\right]\\ d\left[P53^{*}\cdot Bcl2\right]/dt=k_{25}^{+}\left[P53^{*}\right]\left[Bcl2\right]-k_{25}^{-}\left[P53^{*}\cdot Bcl2\right]\\ d\left[Bcl2\cdot Bax\right]/dt=k_{24}^{+}\left[Bcl2\right]\left[Bax\right]-k_{24}^{-}\left[Bcl2\cdot Bax\right] \end{array}$
	$\begin{array}{c} d\left[mitRos\right]/dt=k_{29}^{+}\left[Aqcis\right]\left[Mit\right]\\ d\left[mitPTP^{*}\right]/dt=k_{30}^{+}\left[mitRos\right]\left[mitPTP\right]\\ d\left[Cytc_{\mathrm{mit}}\right]/dt=-k_{26}^{+}\left[Bax\cdot Bak\right]\left[Cytc_{\mathrm{mit}}\right]-k_{31}^{+}\left[mitPTP^{*}\right]\left[Cytc_{\mathrm{mit}}\right]\\ d\left[Cyct\right]/dt=k_{26}^{+}\left[Bax\cdot Bak\right]\left[Cytc_{\mathrm{mit}}\right]-k_{27}^{+}\left[Cytc\right]\left[Casp9\right]\\ \;+k_{27}^{-}\left[Cytc\cdot Casp9\right]+k_{31}^{+}\left[mitPTP^{*}\right]\left[Cytc_{mit}\right]\\ d\left[Cytc\cdot Casp9\right]/dt=k_{27}^{+}\left[Cytc\right]\left[Casp9\right]-k_{27}^{-}\left[Cytc\cdot Casp9\right]\\ \;-k_{28}^{+}\left[Cytc\cdot Casp9\right]\\ d\left[FasL^{*}\right]/dt=k_{32}^{+}\left[Aqcis\right]\left[FasL\right]-k_{33}^{+}\left[FasL^{*}\right]\left[Casp8\right]+k_{33}^{-}\left[FasL^{*}\cdot Casp8\right]\\ d\left[Casp8\right]/dt=-k_{33}^{+}\left[FasL^{*}\right]\left[Casp8\right]+k_{33}^{-}\left[FasL^{*}\cdot Casp8\right]\\ \;-k_{34}^{+}\left[FasL^{*}\cdot Casp8\right] \end{array}$
	$\begin{array}{c} d\left[FasL^{*}\cdot Casp8\right]/dt=k_{33}^{+}\left[FasL^{*}\right]\left[Casp8\right]-k_{33}^{-}\left[FasL^{*}\cdot Casp8\right]-k_{34}^{+}\left[FasL^{*}\cdot Casp8\right]\\ d\left[Casp8^{*}\right]/dt=k_{34}^{+}\left[FasL^{*}\cdot Casp8\right]-k_{35}^{+}\left[Casp8^{*}\right]\left[Casp3\right]+k_{35}^{-}\left[Casp8^{*}\cdot Casp3\right]\\ d\left[Casp8^{*}\cdot Casp3\right]dt=k_{35}^{+}\left[Casp8^{*}\right]\left[Casp3\right]-k_{35}^{-}\left[Casp8^{*}\cdot Casp3\right]\\ \;-k_{36}^{+}\left[Casp8^{*}\cdot Casp3\right]\\ d\left[Apop\right]/dt=k_{11}^{+}\left[Casp9^{*}\right]\left[Casp3^{*}\right]+k_{21}^{+}\left[Casp2^{*}\right]\left[AIF\right]\\ \;+k_{37}^{+}\left[Casp8^{*}\right]\left[Casp3^{*}\right] \end{array}$

### Parameters

Simulation of a differential equation requires parameters for each step of the biochemical reactions. For our model, parameter values were chosen based on previous experimental reports on various apoptosis pathways [26,63]. For parameters that had not been reported in the literature, we adjusted the values to achieve consistency with other parameters and with output through iterative computations using MATLAB. Table 5 and Table 6 show kinetic parameters of the reactions (reaction rate constants) and the initial conditions of the components. The reaction rates are dependent on these concentrations and on biochemical parameters. However, most of the kinetic parameters and initial concentrations (t  =  0) are unknown and subject to parameter estimation. To resolve the problem of a large number of unknown parameters, Zhang et al. [61] and other groups [64-66] used dimensionless concentrations for all concentrations. Because we do not have sufficient experimental data and information in the model, we also used dimensionless concentrations and set the initial conditions to 0 or 1. The pre-expressed components, such as caspase-3 and cytochrome *c* in mitochondria (Cyt.*c*_mit_), were set to 1, and the post-expressed components by stimulus, such as activated caspase-3 and leaked cytochrome *c* in the cytosol (Cyt.*c*_leaked_), were set to 0 at time 0. All values are given in arbitrary units, i.e., dimensionless or relative units. 

**Table 5:** Parameter values adopted in the model

**Reaction rate constants**							
k_1_^+^	1 μM^-1^ s^-1^			k_20_^+^	1 μM^-1^ s^-1^		
k_2_^+^	0.5 μM^-1^ s^-1^			k_21_^+^	1 μM^-1^ s^-1^		
k_3_^+^	1 μM^-1^ s^-1^	k_3_^-^	1 s^-1^	k_22_^+^	1 μM^-1^ s^-1^	k_22_^-^	1 s^-1^
k_4_^+^	1 s^-1^			k_23_^+^	1 s^-1^		
k_5_^+^	1 μM^-1^ s^-1^	k_5_^-^	1 s^-1^	k_24_^+^	1 μM^-1^ s^-1^	k_24_^-^	1 s^-1^
k_6_^+^	1 s^-1^			k_25_^+^	1 μM^-1^ s^-1^	k_25_^-^	1 s^-1^
k_7_^+^	10 μM^-1^ s^-1^	k_7_^-^	0.5 s^-1^	k_26_^+**†**^	10 μM^-1^ s^-1^		
k_8_^+^	1 s^-1^			k_27_^+^	1 μM^-1^ s^-1^	k_27_^-^	1 s^-1^
k_9_^+**†**^	10 μM^-1^ s^-1^	K_9_^-**†**^	0.5 s^-1^	k_28_^+^	1 s^-1^		
k_10_^+**†**^	0.1 s^-1^			k_29_^+^	0.5 μM^-1^ s^-1^		
k_11_^+^	1 μM^-1^ s^-1^			k_30_^+^	0.5 μM^-1^ s^-1^		
k_12_^+**†**^	5 μM^-1^ s^-1^	K_12_^-**†**^	0.0035 s^-1^	k_31_^+^	1 μM^-1^ s^-1^		
k_13_^+**†**^	5 μM^-1^ s^-1^	K_13_^-**†**^	0.0035 s^-1^	k_32_^+^	0.5 μM^-1^ s^-1^		
k_14_^+^	0.5 μM^-1^ s^-1^			k_33_^+^	1 μM^-1^ s^-1^	k_33_^-^	1 s^-1^
k_15_^+^	0.5 μM^-1^ s^-1^			k_34_^+^	1 s^-1^		
k_16_^+^	1 μM^-1^ s^-1^	k_16_^-^	1 s^-1^	k_35_^+^	1 μM^-1^ s^-1^	k_35_^-^	1 s^-1^
k_17_^+^	1 s^-1^			k_36_^+^	1 s^-1^		
k_18_^+^	1 μM^-1^ s^-1^	k_18_^-^	1 s^-1^	k_37_^+^	1 μM^-1^ s^-1^		
k_19_^+^	1 s^-1^						

**†**, cited parameters [44]. Unit of reaction rate constant: M^–1^ s^–1^ for bimolecular reactions, s^–1^ for monomolecular reactions [44].

**Table 6:** All values are in arbitrary units

**Initial conditions**			
OCT2	1	Apoptosis	0
ER	1	Aqcis	0
Calpain	1	Calpain*	0
Caspase-12	1	Caspase-12*	0
Caspase-9	1	Caspase-9*	0
Caspase-3	1	Caspase-3*	0
IAP	1	Caspase-9*·IAP	0
DNA	1	DNA damage	0
ATR	1	ATR*	0
p53	1	p53*	0
AIFmit	1	AIF	0
Bax	1	Bax·Bak	0
Bcl-2	1	p53*·Bcl-2	0
Mit	1	Cyt.*c*·Caspase-9	0
mitPTP	1	mitPTP*	0
Cyt.*c*_mit_	1	Cyt.*c*	0
Caspase-8	1	Caspase-8*	0
FasL	1	FasL*	0
Caspase-2	1	Caspase-2*	0
Ca^2+^	0	Caspase-8*·Caspase-3	0
Ca^2+^·Calpain	0	FasL*·Caspase-8	0
Calpain*·Caspase-12	0	ATR*·p53	0
mitRos	0	Caspase-3*·IAP	0
Caspase-12*·Caspase-9	0	Caspase-9*·Caspase-3	0
p53*·Caspase-2	0	p53*·Bax	0
Bcl-2·Bax	0		

*, activated components.

**Figure 8:**
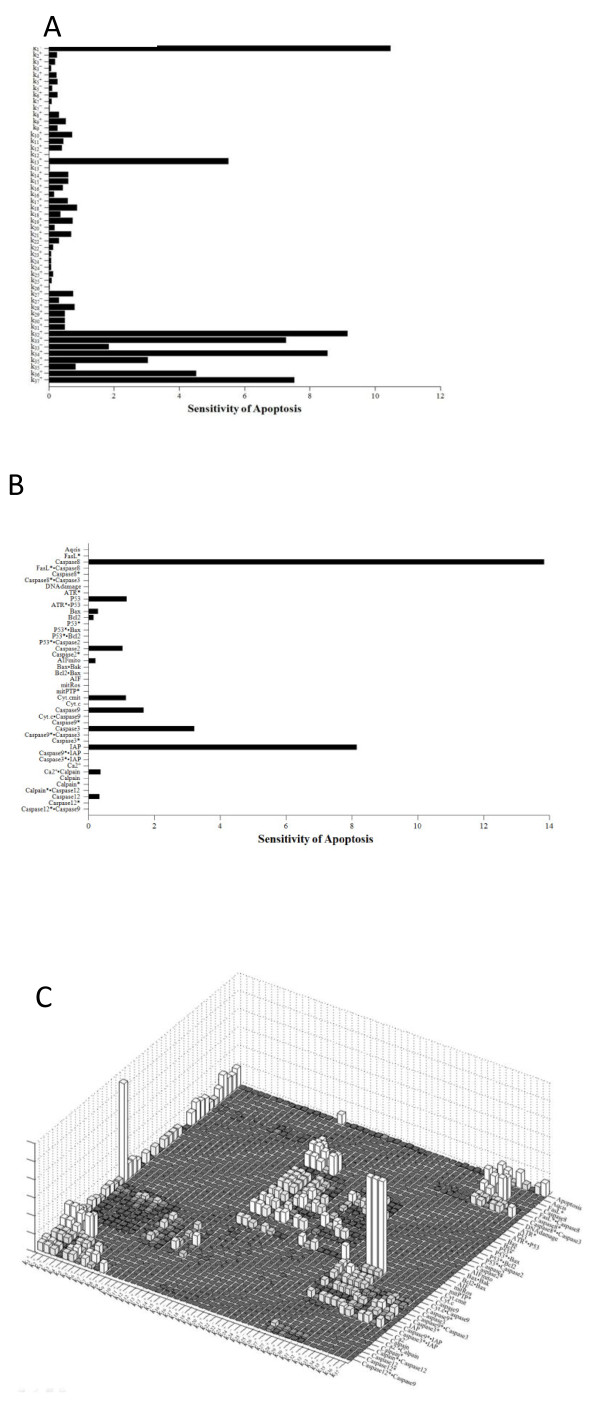
**Sensitivity analyses of rate constants and initial conditions for the entire model.** (**A**) Sensitivity of each rate constant for apoptosis. (**B**) Sensitivity of each initial condition for apoptosis. (**C**) Sensitivity of each rate constant for whole components.

Sensitivity analysis of the model parameters and initial conditions of the model variables were performed using SimBiology in MATLAB. As is shown in Figure 8, sensitivity analysis was carried out with respect to rate constants, initial conditions of model variables, and both rate constants and initial conditions, respectively. Among the rate constants, the parameter k1 exhibited a relatively high sensitivity (Figure 8A). Sensitivity analysis of initial conditions of the model variables (Figure 8B) showed that the level of apoptosis was more sensitive to the initial conditions in terms of Caspase8 and IAP (Figure 8B). Figure 8C shows the sensitivity of each rate constant for the whole model. For rate constants that had relatively high sensitivities, we tested the robustness of the parameters by perturbing the values within +/− 30%. Table 7 shows the results of the perturbation test. In all cases tested, the level of apoptosis changed slightly, within +/−10% according to the variation in the chosen parameter, except for parameters, k1 and k13, demonstrating the overall robustness of the simulation results with respect to parameter perturbations. 

**Table 7:** Effects of parameters on apoptosis

**Selected parameters (Higher sensitive parameters in sensitivity analysis)**	**+30% parameter value**		**−30% parameter value**	
	**Parameter range**	**Apoptosis level (0.22512)**	**Parameter range**	**Apoptosis level (0.22512)**
k_1_^+^ = 1 μM^-1^ s^-1^	k_1_^+^ = 1.3	0.24831 (+10.3%)	k_1_^+^ = 0.7	0.19202 (−14.7%)
k_13_^+^ = 5 μM^-1^ s^-1^	k_13_^+^ = 6.5	0.19674 (−12.6%)	k_13_^+^ = 3.5	0.27136 (+20.54%)
k_14_^+^ = 0.5 μM^-1^ s^-1^	k_14_^+^ = 0.65	0.23067 (+2.46%)	k_14_^+^ = 0.35	0.2172 (−3.52%)
k_15_^+^ = 0.5 μM^-1^ s^-1^	k_15_^+^ = 0.65	0.23067 (+2.46%)	k_15_^+^ = 0.35	0.2172 (−3.52%)
k_29_^+^ = 0.5 μM^-1^ s^-1^	k_29_^+^ = 0.65	0.22952 (+1.95%)	k_29_^+^ = 0.35	0.21911 (−2.66%)
k_30_^+^ = 0.5 μM^-1^ s^-1^	k_30_^+^ = 0.65	0.22952 (+1.95%)	k_30_^+^ = 0.35	0.21911 (−2.66%)
k_31_^+^ = 1 μM^-1^ s^-1^	k_31_^+^ = 1.3	0.22952 (+1.95%)	k_31_^+^ = 0.7	0.21911 (−2.66%)
k_32_^+^ = 0.5 μM^-1^ s^-1^	k_32_^+^ = 0.65	0.23424 (+4.05%)	k_32_^+^ = 0.35	0.20999 (−6.72%)
k_33_^+^ = 1 μM^-1^ s^-1^	k_33_^+^ = 1.3	0.232 (+3.05%)	k_33_^+^ = 0.7	0.21389 (−4.98%)
k_34_^+^ = 1 s^-1^	k_34_^+^ = 1.3	0.2392 (+6.25%)	k_34_^+^ = 0.7	0.19967 (−11.3%)
k_36_^+^ = 1 s^-1^	k_36_^+^ = 1.3	0.238 (+5.72%)	k_36_^+^ = 0.7	0.20434 (−9.23%)

## Competing interests

The authors declared that they have no competing interests.

## Authors’ contributions

JYH designed the model and performed the simulations, and wrote the manuscript. GHK and JWK assisted modeling and writing of the manuscript. SSK assisted modeling. ES and KHC helped the simulations and reviewed the manuscript. EBS designed/supervised the work and reviewed the manuscript. All authors read and approved the final manuscript.
